# *Anthemosoma garnhami* in an HIV-Infected Man from Zimbabwe Living in South Africa

**DOI:** 10.3201/eid2707.204759

**Published:** 2021-07

**Authors:** David Stead, Desiree du Plessis, Lisa Ming Sun, John Frean

**Affiliations:** Walter Sisulu University, Mthatha, South Africa (D.F. Stead);; Cecilia Makiwane Hospital, East London, South Africa (D.F. Stead);; National Institute for Communicable Diseases, Johannesburg, South Africa (D. du Plessis, L. Ming Sun, J. Frean);; University of the Witwatersrand, Johannesburg (J. Frean)

**Keywords:** Africa, Anthemosoma garnhami, HIV/AIDS and other retroviruses, murine, opportunistic infections, parasites, piroplasm, recrudescence, rodents, tick-borne diseases, vector-borne infections, viruses

## Abstract

An HIV-positive man from Zimbabwe living in South Africa sought treatment for multiple clinical signs, including fever, weight loss, anemia, and splenomegaly. We identified in his blood an African rodent piroplasm, *Anthemosoma garnhami*, related to *Babesia* species. This finding extends the known geographic and host range of *A. garnhami*.

A 24-year-old man from Zimbabwe who had been living in East London, South Africa, for 13 years attended a primary health care clinic in East London complaining of a 3-month period of generalized body pains, drenching night sweats, and weight loss. He had no notable previous medical history. The attending nurse diagnosed HIV infection by rapid test, collected sputum for an Xpert MTB/RIF test (Cepheid, https://www.cepheid.com), and requested blood screening as preparation before initiating combination antiretroviral therapy. Malaria-like objects found on the blood smear prompted referral for specialist opinion at Cecilia Makiwane Hospital in Mdantsane, South Africa. This case report was approved by the Human Research Committee of the Faculty of Health Sciences, Walter Sisulu University, Mthatha, South Africa (protocol no. 126/2020). The patient granted written informed consent for publication of the case report.

The patient shared a house with another adult (no animals) and worked as a construction laborer. Four months before seeking treatment, he returned from a 2-month home visit to Masvingo Province in Zimbabwe. He did not recall tick bites but reported that goats and cattle lived in the village he visited. 

At hospital admission, the patient was wasted (40 kg), generally weak, afebrile, and markedly pale; he had oral candidiasis. His enlarged, smooth, nontender spleen was palpable to ≈10 cm below the costal margin in the midclavicular line. No other findings were remarkable. Laboratory results (Table) showed evidence of likely hypersplenism-related pancytopenia, hemolysis, mildly raised transaminases, and advanced HIV infection. The abnormal blood smear showed intraerythrocytic parasites, initially thought to be malarial. However, concurrent rapid malaria antigen tests were negative, and the smears and whole blood sample were sent to a national parasitology reference laboratory for further assessment. On the basis of microscopic examination of Giemsa-stained blood smears (Figure), we diagnosed babesiosis accompanied by hemolytic anemia. 

**Table:** Laboratory findings over time for an HIV-positive patient from Zimbabwe living in South Africa who was infected with the rodent piroplasm *Anthemosoma garnhami*

Laboratory test	Dates					Reference values
	2019 May 31	2019 Jun 7*	2019 Jun 20	2020 Aug 31†	2020 Nov 11	
Hemoglobin, g/L	45	40	92	59	96	132–173
Mean cell volume, × 10^15^/L	82	73	84	81	84	80–99
Leukocyte count, × 10^9^/L	0.34	2.4	3.6	2.2	3.8	4–11
Platelet count, × 10^9^/L	123	140	184	56	103	137–373
Reticulocytes, %		5.6				0.5–1.5
Creatinine, mg/dL		0.79				0.5–1.5
Total bilirubin, mg/dL		0.41				0.3–1.0
Aspartate transaminase, U/L		111				10–30
Alanine transaminase, U/L	19	47				10–40
Lactate dehydrogenase, U/L		922				100–200
Haptoglobin, g/L		<0.1				0.3–2.0
CD4, cells/mm^3^	70			195		500–1,200
HIV ELISA	Positive					
Sputum Xpert MTB/RIF‡	Negative					

*Date of treatment initiation. †Date of recrudescence and retreatment initiation. ‡*Mycobacterium tuberculosis* and rifampin resistance testing (Cepheid, https://www.cepheid.com).

**Figure:**
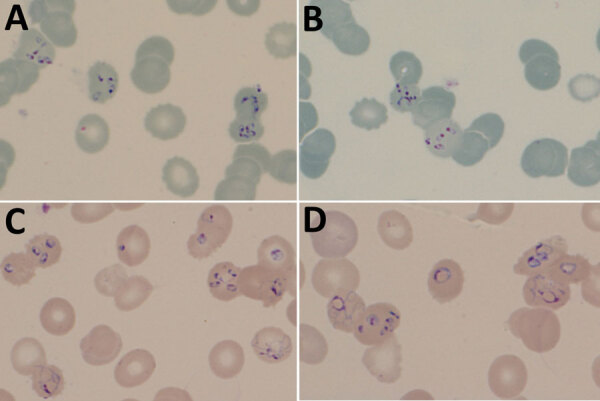
Thin blood film photographs showing *Babesia*-like early tetrads (panels A, B) and pleiomorphic later-stage parasites (panels C, D) in an HIV-positive patient from Zimbabwe living in South Africa. The multiply infected erythrocytes and unusual morphology suggested nonmalaria parasites and were later determined to be the rodent piroplasm *Anthemosoma garnhami*, related to *Babesia* spp. Slides stained with 10% Giemsa, pH 7.2, for 20 min; original magnification ×1,000.

We started the patient on a 10-day course of oral clindamycin and quinine (each 600 mg every 8 h). Blood transfusion was not needed. After 2 weeks, all symptoms improved markedly; splenomegaly was reduced to 5 cm below the costal margin and hemoglobin substantially improved. We subsequently initiated tenofovir, emtricitabine/efavirenz combination antiretroviral therapy, and trimethoprim/sulfamethoxazole prophylaxis; the patient responded well in the hospital antiretroviral unit.

DNA extracted from anticoagulated whole blood tested negative for *Plasmodium* spp. by multiplex real-time PCR for malaria. We used a nested conventional PCR assay for the *Babesia* species 18S RNA gene (*1*) and applied bidirectional Sanger sequencing to the 400-bp product, showing sequences shared across members of order Piroplasmida. We used PCR with 18S RNA universal primers to refine this result. (*2*). The ≈1,700 bp product sequence (GenBank accession no. MW276138) had 99.15% identity and a subsequent sequence (accession no. MW276139) from a recrudescence, 99.03% identity with the murine piroplasm *Anthemosoma garnhami* (accession no. MH093637.1; Appendix Figure).

The patient returned for treatment 14 months later. He had not traveled outside of South Africa since his initial treatment. He was again pale and had an enlarged spleen. His hemoglobin was 59 g/L, and we again observed intraerythrocytic piroplasms on the blood smear. His CD4 count was now 195 cells/mm^3^ and HIV viral load 304 copies/mL. He was admitted for intravenous clindamycin and oral quinine (each 600 mg every 8 h) as part of a 6-week treatment plan. The patient responded well clinically and hematologically to treatment (Table).

*A. garnhami* is an erythrocytic murine parasite, first described in spiny mice (*Acomys percivali*) in Ethiopia in 1969 (*3*). Because it shares characteristics with Haemosporidia and Piroplasmida, its classification was long debated, but on the basis of ribosomal RNA analysis of archived *A. garnhami* samples, it was finally assigned to the piroplasms, as the sole species of the family Anthemosomatidae (*3*). The parasite was identified again in 2 different rodent species in Namibia (*4**,**5*). Ixodid ticks serve as vectors of piroplasms and therefore are likely vectors for *A. garnhami*; experiments failed to demonstrate transmission by several tick and mosquito species (*3*). *A. garnhami* is closely related to the babesids in the Piroplasmida order, hence the similar microscopic appearance and the good clinical response in this case to clindamycin and quinine, drugs used to treat *Babesia* spp. Babesiosis in immunocompromised patients, including those with HIV, is more severe and more likely to recur (*6*). The recrudescing clinical course of this *A. garnhami* infection was probably exacerbated by the patient’s advanced HIV disease. 

Our report establishes a likely epizootologic similarity between *A. garnhami* and *Babesia* spp., suggesting the potential for *A. garnhami* to cause zoonotic infections in humans. Although babesiosis in domestic animals is common in Africa and *B. microti* has been found in nonhuman primates in East Africa (*7*), only single reports from southern Africa (*8*) and Equatorial Guinea (*9*) have described human *Babesia* spp. infections. The conjunction of high concentrations of ticks, animals, malaria, and HIV-infected humans in Africa make it possible for piroplasm infections to be misdiagnosed as malaria, which poses potentially serious clinical consequences for immunocompromised patients. 

Appendix Additional information about the *Anthemosoma garnhami* parasite discovered in an HIV-positive man from Zimbabwe living in South Africa.
