# Supplementary material for: Anthemosoma garnhami in an HIV-Infected Man from Zimbabwe Living in South Africa
Source: Emerg Infect Dis. 2021 Jul;27(7):1991–3. doi: 10.3201/eid2707.204759 (PMC8237893; doi:10.3201/eid2707.204759)
Supplement: Appendix — Additional information about the Anthemosoma garnhami parasite discovered in an HIV-positive man from Zimbabwe living in South Africa. [file 20-4759-Techapp-s1.pdf]

Article DOI: https://doi.org/10.3201/eid2707.204759

Anthemosoma garnhami in an HIV-Infected Man from Zimbabwe Living in South Africa

Appendix

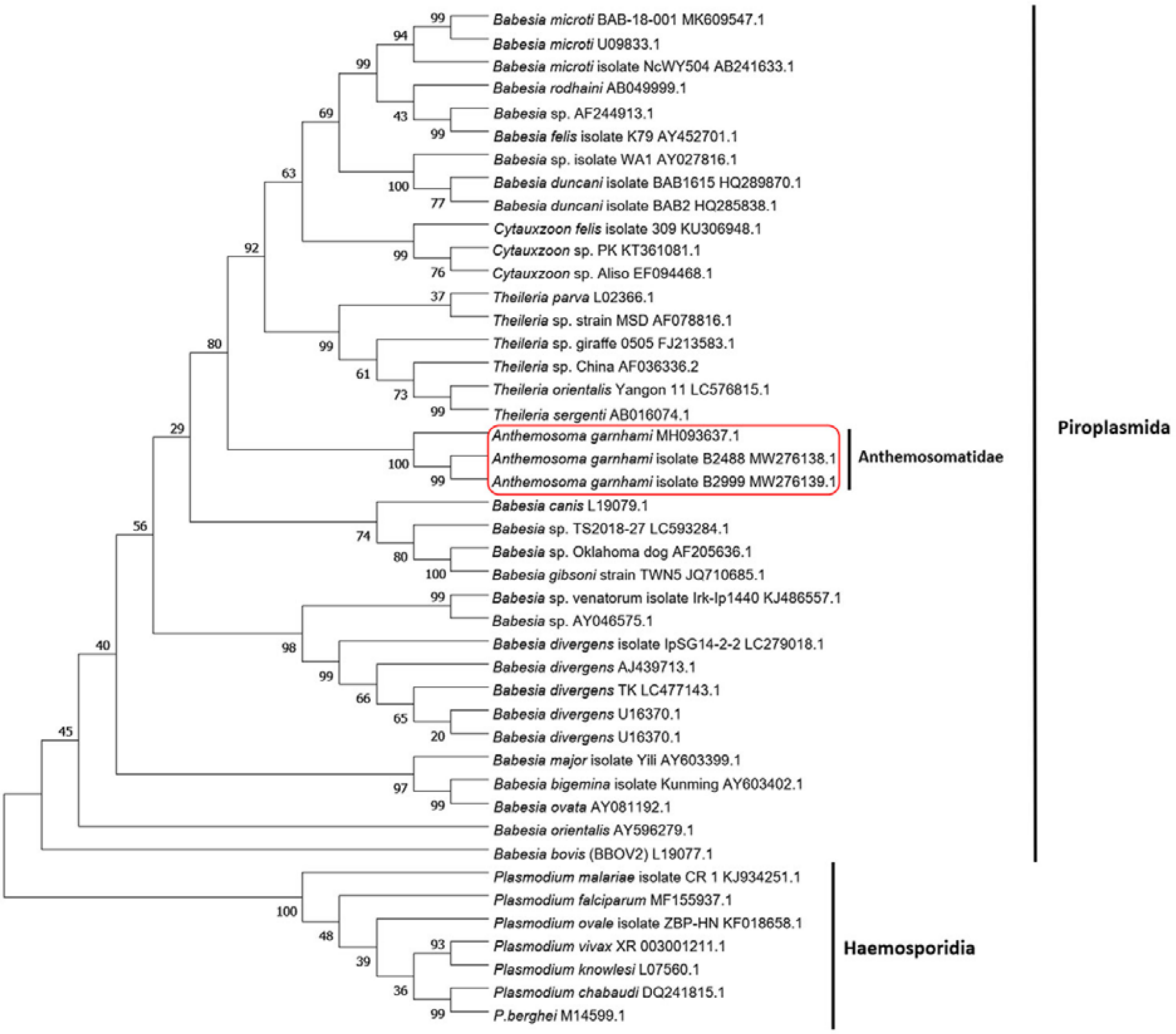

Appendix Figure. Molecular phylogeny of piroplasms based on the nuclear 18S RNA gene. The phylogenetic position of *Anthemosoma garnhami* sequences from the original and recrudescent infections described in this report are shown in the box. The analysis included 44 nucleotide sequences (numbers in parentheses): 35 from other piroplasms, and 7 *Plasmodium* sequences, as outgroup. The evolutionary history was inferred by using the maximum likelihood method based on the Tamura-Nei model (*1*,*2*). The tree with the highest log likelihood (–4698.01) is shown. Initial

tree(s) for the heuristic search were obtained automatically by applying neighbor-join and BioNJ algorithms to a matrix of pairwise distances estimated using the maximum composite likelihood approach, and then selecting the topology with superior log likelihood value. The tree is drawn to scale, with branch lengths measured in the number of substitutions per site. All positions containing gaps and missing data were eliminated. There were a total of 690 positions in the final dataset. Evolutionary analyses were conducted in MEGA7 (*3*).

References

1. Tamura K, Nei M. Estimation of the number of nucleotide substitutions in the control region of mitochondrial DNA in humans and chimpanzees. Mol Biol Evol. 1993;10:512–26. PubMed
2. Tamura K, Battistuzzi FU, Billing-Ross P, Murillo O, Filipski A, Kumar S. Estimating divergence times in large molecular phylogenies. Proc Natl Acad Sci U S A. 2012;109:19333–8. PubMed https://doi.org/10.1073/pnas.1213199109
3. Kumar S, Stecher G, Tamura K. MEGA7: Molecular Evolutionary Genetics Analysis version 7.0 for bigger datasets. Mol Biol Evol. 2016;33:1870–4. PubMed https://doi.org/10.1093/molbev/msw054
